# The Role of Exosomes in Pancreatic Ductal Adenocarcinoma Progression and Their Potential as Biomarkers

**DOI:** 10.3390/cancers15061776

**Published:** 2023-03-15

**Authors:** Sheng-Kai Hsu, Mahendra Jadhao, Wei-Ting Liao, Wen-Tsan Chang, I-Ling Lin, Chien-Chih Chiu

**Affiliations:** 1 Department of Biotechnology, Kaohsiung Medical University, Kaohsiung 807, Taiwan; 2 Department of Medical Laboratory Science and Biotechnology, Kaohsiung Medical University, Kaohsiung 807, Taiwan; 3 Department of Cancer Biology, University of Cincinnati College of Medicine, Cincinnati, OH 45267, USA; 4 Division of General and Digestive Surgery, Department of Surgery, Kaohsiung Medical University Hospital, Kaohsiung 807, Taiwan; 5 Department of Surgery, School of Medicine, College of Medicine, Kaohsiung Medical University, Kaohsiung 807, Taiwan; 6 Center for Cancer Research, Kaohsiung Medical University Hospital, Kaohsiung Medical University, Kaohsiung 807, Taiwan; 7 Department of Laboratory Medicine, Kaohsiung Medical University Hospital, Kaohsiung 807, Taiwan; 8 Department of Medical Research, Kaohsiung Medical University Hospital, Kaohsiung 807, Taiwan; 9 Department of Biological Sciences, National Sun Yat-sen University, Kaohsiung 804, Taiwan; 10 The Graduate Institute of Medicine, Kaohsiung Medical University, Kaohsiung 807, Taiwan

**Keywords:** pancreatic ductal adenocarcinoma, tumor microenvironment, exosome, biomarker, tumor progression

## Abstract

**Simple Summary:**

Pancreatic ductal adenocarcinoma (PDAC) is an aggressive and lethal malignancy with a dismal five-year survival rate. Despite remarkable improvements of cancer therapeutics in recent years, patients with PDAC barely benefit from them due to late diagnosis of the disease. Exosomes, small extracellular vesicles with a diameter of approximately 30 to 150 nm, play a significant role in cell–cell communication via cargo delivery (e.g., proteins, lipids, and nucleic acids) among heterogeneous populations. Emerging studies have suggested that exosomes with specific surface markers or contents are capable of discriminating PDAC patients from health individuals. Because detectable in several body fluids, such as blood, urine and saliva, exosomes are considered as promising liquid biopsies for early detection and disease monitoring. In this review, we shed light on the involvement of exosomes and their cargos in PDAC progression and their feasibility as diagnostic and prognostic biomarkers. In addition, limitations and urgent problems required further investigation are also discussed in our review.

**Abstract:**

Pancreatic ductal adenocarcinoma (PDAC), the most common pancreatic malignancy, is an aggressive and lethal cancer with a dismal five-year survival rate. Despite remarkable improvements in cancer therapeutics, the clinical outcome of PDAC patients remains poor due to late diagnosis of the disease. This highlights the importance of early detection, wherein biomarker evaluation including exosomes would be helpful. Exosomes, small extracellular vesicles (sEVs), are cell-secreted entities with diameters ranging from 50 to 150 nm that deliver cellular contents (e.g., proteins, lipids, and nucleic acids) from parent cells to regulate the cellular processes of targeted cells. Recently, an increasing number of studies have reported that exosomes serve as messengers to facilitate stromal-immune crosstalk within the PDAC tumor microenvironment (TME), and their contents are indicative of disease progression. Moreover, evidence suggests that exosomes with specific surface markers are capable of distinguishing patients with PDAC from healthy individuals. Detectable exosomes in bodily fluids (e.g., blood, urine, saliva, and pancreatic juice) are omnipresent and may serve as promising biomarkers for improving early detection and evaluating patient prognosis. In this review, we shed light on the involvement of exosomes and their cargos in processes related to disease progression, including chemoresistance, angiogenesis, invasion, metastasis, and immunomodulation, and their potential as prognostic markers. Furthermore, we highlight feasible clinical applications and the limitations of exosomes in liquid biopsies as tools for early diagnosis as well as disease monitoring. Taking advantage of exosomes to improve diagnostic capacity may provide hope for PDAC patients, although further investigation is urgently needed.

## 1. Introduction

Pancreatic ductal adenocarcinoma (PDAC) is a malignancy originating from the cells lining pancreatic ducts, which are responsible for the transport of digestive enzymes. PDAC, accounting for 90% of all pancreatic malignancies, is an intractable cancer with a dismal five-year survival rate of 11% [1]. According to the numbers of new cancer cases and deaths estimated by the American Cancer Society in 2023, pancreatic cancer (PC) is the third leading cause of cancer-related mortality in the U.S. [2]. If this trend continues, PC is predicted to become second common cancer-associated death in 2040 [3]. The poor clinical outcome is primarily attributed to late diagnosis of the disease. It is estimated that 80% of PC patients are diagnosed at advanced or metastatic stages [1]. On the basis of the guidelines of the National Comprehensive Cancer Network, surgical resection is the only curative approach, and some research has indicated that the five-year survival rate can reach 58% after curative resection. Nevertheless, only 20% of PDAC patients are eligible for resection at the time of diagnosis [4,5,6]. Notably, according to cancer statistics, the five-year survival rate is 42% for patients with localized PDAC, and it dramatically drops to 14% and 3% for those with locally advanced disease or distant metastasis, respectively [1]. A previous study indicated that lymph node spread occurs in 30% of patients with tumors less than 1 cm, and distant metastasis is observed in 10% of those patients [7]. These findings represent the early progression of PDAC. Other statistics conducted by the Japan Pancreas Society revealed that the five-year survival rates of Union for International Cancer Control (UICC) stage 0, Ia, and Ib PDAC are 85.8%, 68.7%, and 59.7%, respectively, showing a relatively favorable prognosis; however, these populations account for only 12% of all PDAC cases [8]. Taken together, these findings highlight the importance of early detection of PDAC.

Exosomes, small extracellular vesicles (sEVs) with a diameter of approximately 30 to 150 nm released from parental cells, play a significant role in cell–cell communication via cargo delivery (e.g., proteins, lipids, and nucleic acids) among heterogeneous cell populations [9]. Accumulating studies have suggested that exosomes with specific markers are capable of discriminating patients with PDAC from healthy subjects (HSs) and can even be detected in circulation prior to lesions being detectable on magnetic resonance imaging (MRI) [10,11]. In general, cancer cells tend to secrete more exosomes than normal cells, and exosomal cargos are involved in processes related to cancer progression, including angiogenesis, metastasis, drug resistance, and immunosuppression [12,13,14]. Owing to their presence in almost all body fluids (e.g., blood, urine, saliva, and pancreatic juice), exosomes detected in liquid biopsy have potential utility for early detection and disease monitoring [15].

In this review, we first introduce the biological significance of exosomes, including the biogenesis, secretion, uptake, and bioactivities of exosomal cargos. Furthermore, we shed light on the involvement of exosomes and their cargos within the tumor microenvironment (TME) in PDAC progression (e.g., chemoresistance, angiogenesis, invasion, metastasis, and immune surveillance escape) and their feasibility as diagnostic and prognostic biomarkers. Finally, we discuss further clinical implications, including screening and early detection strategies, engineering exosomes as therapeutics, and the limitations as well as urgent problems related to the use of exosomes as potential biomarkers that need further investigation.

## 2. Biological Characteristics of Exosomes

sEVs are small-lipid-bilayer-containing molecules secreted by various types of cells. sEVs are a group of heterogeneous endosome/plasma membrane-derived vesicles and are primarily considered cellular waste disposal entities. On the basis of size, biogenesis, and secretory mechanisms, sEVs are further classified as exosomes, microvesicles, and apoptotic bodies [16]. Exosomes are the smallest sEVs, measuring <100 nm, and are formed from multiple endosomes by exocytic budding of the plasma membrane [17]. In recent years, sEVs have gained more attention for their role in cellular communication and pathophysiological conditions such as cancer [18]. Among sEVs, exosomes in particular have gained attention, and their ability to carry cargo from parent cells is a major feature underlying this interest. Exosome cargos mainly comprise parent cell components, such as proteins, lipids, and nuclear acids, which are further delivered to recipient cells, influencing several cellular functions [19]. Increased understanding of the biological activity of exosomal contents and the ability of exosomes to deliver cargo may not only shed light on disease progression but also provide an opportunity for therapeutic approaches. In this section, we briefly discuss the journey of exosomes from their biogenesis to cellular uptake and categories of exosomal cargos.

### 2.1. Journey of Exosomes: Biogenesis, Secretion, and Uptake

**Figure 1:**
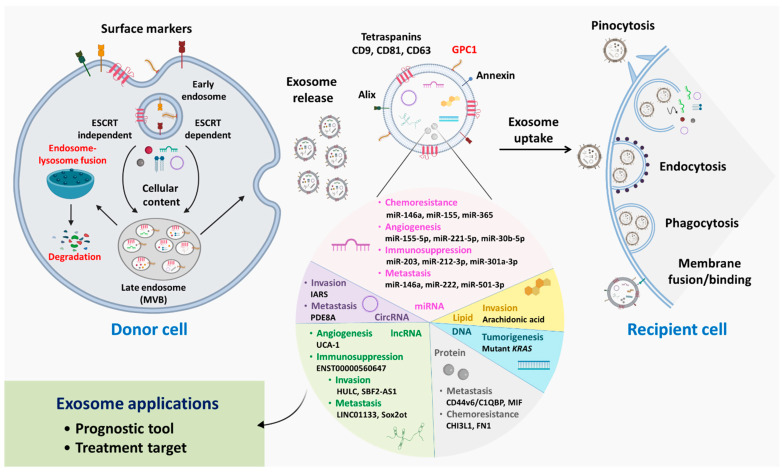
Insights into exosome biogenesis, cargo packaging, and cellular uptake. Inward budding of the cellular membrane facilitates early endosome formation; during this process, cell surface markers/receptors are loaded within endosomal bodies, which are later present on the exosomal surface. During maturation, cellular contents, including proteins, lipids, DNA, circRNAs, miRNAs, lncRNAs, and mRNAs, are loaded into endosomes by ESCRT-dependent or ESCRT-independent mechanisms, forming late endosomes/MVBs. Late endosomes may undergo recycling following fusion with lysosomes, degrading endosomal contents; otherwise, late endosomes are moved to the cell membrane, resulting in membrane fusion and exosome release from cells. Exosomal entry into recipient cells is directed by one of several mechanisms, such as pinocytosis, endocytosis, phagocytosis, and membrane binding/fusion, depending on the cellular receptors and exosomal surface molecules present. Exosomal cargos and their effects on PDAC progression are summarized in the pie chart. Overall, evaluation of exosomal contents may be used in clinical applications for disease prognosis prediction, and exosomes may be employed as antagonist delivery systems for treatment.

The biogenesis process of exosomes differentiates them from other sEVs. Exosome biogenesis is a complex process involving several steps, such as processing of late endosomes/multivesicular bodies (MVBs) to form intraluminal vesicles (ILVs) in MVBs, transportation of MVBs to the cellular plasma membrane, fusion with the plasma membrane, and release. During the process of ILV formation in MVBs, several proteins and cytoplasmic contents along with a considerable portion of nucleic acids (DNA and RNA) are packaged within ILVs, constituting exosome cargo; the ILVs fuse with the plasma membrane and are released as exosomes [20]. On the other hand, lysosomal fusion may result in late endosome/MVB clearance. Several molecules are involved in the processing and release of exosomes, and the biogenesis mechanism can be either endosomal sorting complex required for transport (ESCRT)-dependent or ESCRT-independent [21] (Figure 1). Apart from conventional Rab GTPase-mediated MVB processing and exosome release, physiological conditions such as hypoxic stress, heat shock, DNA damage, and intracellular calcium and thrombin accumulation influence exosome release [22,23,24,25].

As described previously, exosomes are released by parent cells as part of the biogenesis process. Once released, exosomes either target proximal cells or diffuse systemically to distant locations, which explains their omnipresence in body fluids. Exosomal interaction with target cells depends on several factors regulating physiological mechanisms; for example, phagocytosis is facilitated by exosomal surface markers. The presence of opsonins such as phosphatidylcholine, phosphatidylserine, lactadherin, actins, and dynamin 2 promotes exosome phagocytosis by phagocytic cells [26]. Exosomal phosphatidylserine has also been reported to activate micropinocytosis, in which actin-filament-driven plasma membrane protrusions form a pocket and internalize exosomes [27]. Endocytosis is a common process of exosome internalization among most cells. Endocytosis may be clathrin-, caveolin-, or lipid-raft-mediated, and the presence of these individual molecules mediates exosomal internalization through endosome formation [28]. Membrane fusion is a less complex process in which the exosomal lipid bilayer and cell membrane fuse with each other, forming a hemi-fusion stalk. Ultimately, stalk expansion causes the formation of the fusion pore, leading to mixing of cellular and exosomal hydrophobic cores. The fusion process is mainly driven by Rab and soluble N-ethylmaleimide-sensitive-factor attachment protein receptor (SNARE) proteins [21,29]. Once exosomes enter recipient cells, the exosomal cargo is released, and this cargo further influences and regulates key cellular processes (Figure 1).

### 2.2. Exosomal Cargos and Their Bioactivities

Once considered cellular waste disposal bodies, exosomes have come to be well-studied cellular entities that can serve as disease biomarkers and drug carriers, and exosomal cargos are important in these applications. Exosomes are often referred to as miniature versions of the cells from which they originate, containing nucleic acids, proteins, lipids, and other cytoplasmic contents belonging to their parental cells. However, exosomes lack protein contents from the nucleus, mitochondria, Golgi complex, and endoplasmic reticulum [30]. Recently, extensive research to identify the contents of exosomes has been performed, and the information has been archived in several online databases. Vesiclepedia, Evpedia, and ExoCarta are web-based databases providing information on exosomal contents as well as isolation and characterization methodologies [31,32,33]. A total of 1116 lipids, 9769 proteins, 2838 miRNAs, and 3408 mRNAs have been identified and reported in the abovementioned databases, which are being updated constantly. Tubulins, actins, and associated binding proteins comprise large proportions of the exosomal protein content [30]. In addition, proteins involved in various functional events are also present in exosomes. For example, the protein content of exosomes can consist of fusion-, penetration-, and invasion-associated CD markers/tetraspanins, such as CD81, CD82, CD9, and CD3; antigen-presentation- and stress-response-associated proteins, such as MHC I, HSP70, and HSP90; MVB-creation- and exosome-release-associated molecules, such as TSG101 and Alix; and membrane-fusion- and transport-associated proteins, such as Rab and Annexin [34,35]. Several lipids are also present in exosomes, mainly ceramides, sphingomyelins, cholesterols, phosphatidyl-serine, and some fatty acids (saturated) [36]. Overall, the enrichment of protein and lipid contents in exosomes highlights the active exosomal component sorting mechanism.

The nucleic acid content in exosomes is highly enriched in genomic and/or mitochondrial DNA, mRNAs, and noncoding RNAs (ncRNAs) [37]. Noncoding transcripts that aid protein synthesis without undergoing translation themselves are known as ncRNAs. It is estimated that ncRNAs account for approximately 98% of the mammalian transcriptome; they are divided into short ncRNAs (length < 200 nucleotides) and long ncRNAs (lncRNAs, length > 200 nucleotides) [38,39]. To date, a number of ncRNAs that participate in cellular processes have been identified. For instance, ncRNAs are involved in the mRNA translation machinery, and ribosomal RNAs (rRNAs), transfer RNAs (tRNAs), microRNAs (miRNAs), lncRNAs, circular RNAs (circRNAs), and small nuclear RNAs (snRNAs) have been identified to be related to cancer and its progression [38]. Abundant intracellular expression and omnipresence in the cell cytoplasm leads to exosomal packaging of these ncRNAs, which are further released by tumor cells and delivered to the tumor microenvironment (TME), where they can enact their target function. Hence, in this review, we emphasize the impact of exosomal miRNAs, lncRNAs, and circRNAs on PDAC progression.

#### 2.2.1. microRNAs (miRNAs)

miRNAs are single-stranded ncRNAs of 18–25 nucleotides in length that are endogenous in nature. miRNAs target mRNAs, and miRNA–mRNA interactions result in either mRNA degradation or mRNA translation inhibition, leading to gene silencing [40,41]. miRNAs are a major cargo of exosomes, and they can induce the effects they have in parent cells (mainly mRNA translation regulation) after entering recipient cells [42]. Exosomal miRNAs have been reported to elicit persistent effects in recipient cells, influencing key cellular processes such as proliferation, angiogenesis, metastasis, hematopoiesis, carcinogenesis, and regulation of several other diseases [43,44,45,46,47,48,49,50,51]. Several studies have demonstrated elevated exosome production and secretion by cancerous cells compared to neighboring noncancerous cells. Tumor-cell-secreted exosomes tend to carry tumor-specific miRNAs, which might serve as biomarkers for predicting disease prognosis. Notably, Yang et al. performed a meta-analysis and successfully demonstrated that circulating exosomal miRNAs can accurately identify healthy and diseased individuals among prostate cancer patients [52]. Similarly, accumulating studies have revealed that exosomal miRNA profiling of body fluids (e.g., serum, urine, and pancreatic juice) can be used to discriminate PDAC patients from HSs, highlighting the utility of exosomal miRNA as a disease biomarker [53,54,55,56].

#### 2.2.2. Long Noncoding RNAs (lncRNAs)

Recent evidence has shown that lncRNAs play a significant role in several cellular events, including modulation of transcription, regulation of mRNA stability, and posttranscriptional alteration [57]. lncRNAs in exosomes are released following internalization, after which they affect cellular processes leading to cancer progression. Recent studies have demonstrated the ability of lncRNAs to sponge miRNAs, which influences the ability of miRNAs to target mRNAs and regulate protein expression [58,59]. Furthermore, several lncRNAs have been reported to be involved in PDAC progression and to serve as prognostic indicators. For instance, lncAFAP1-As1 induces lymph node (LN) invasion and perineural invasion (PNI), and elevated lncAFAP1-As1 levels correlate with local recurrence and metastasis after resection [60].

#### 2.2.3. Circular RNAs (circRNAs)

In addition to miRNAs and lncRNAs, other RNAs, such as circRNAs and linear RNAs, can be exosomal cargos. circRNAs play a significant role in cancer progression. Furthermore, their structure endows circRNAs with high stability and protection against RNase R-mediated and exonuclease-mediated degradation within exosomes, giving circRNAs advantages over less-stable linear RNAs, which are easily degraded by RNases and exonucleases. In fact, even if serum samples are placed at room temperature for up to 24 h, the level of circRNAs changes only minimally [61]. Recent studies have suggested that exosomal circRNAs participate in PDAC progression by functioning as sponges to sequester miRNAs, and their levels are indicative of patient prognosis [62,63]. In summary, some exosomal circRNAs are promising prognostic indicators and/or therapeutic targets.

## 3. Involvement of Exosomes in the Crosstalk of the PDAC TME

The pathological characteristics of PDAC are desmoplastic reactions and immunosuppression within the TME [64,65]. Apart from malignant cells, the PDAC TME consists of various cell types (e.g., immune cells and stromal cells) and acellular components (e.g., extracellular matrix (ECM)) [66]. Importantly, the crosstalk of these components within the TME considerably contributes to PDAC tumorigenesis. Once considered cellular waste disposal bodies, exosomes and their contents are now well-studied cellular entities that can be employed as disease biomarkers and have been identified as key players in PDAC progression [19,67]. In this section, we elucidate the involvement of exosomal cargos in processes related to PDAC progression, including chemoresistance, angiogenesis, invasion, metastasis, and immunomodulation, highlighting their promise in diagnostic approaches and as therapeutic targets (Figure 1).

### 3.1. Chemoresistance

Because of late diagnosis, 80% of PDAC patients are ineligible for surgery and require systemic chemotherapy [5]. In particular, gemcitabine (GEM)-based regimens are the first-line treatment for these patients; unfortunately, most patients develop chemoresistance [4,68]. Growing evidence suggests that exosomal cargos are involved in GEM resistance and may be predictive biomarkers that physicians can use to rapidly evaluate patient response to GEM. Macrophages are crucial in inflammation and tissue homeostasis and are mainly characterized by two phenotypes: M1 and M2. The former has proinflammatory and antitumor effects and functions in tissue damage, while the latter has anti-inflammatory and protumor effects and participates in tissue repair [69,70]. Recent studies have revealed the role of M2-macrophage-derived exosomal cargo in chemoresistance. Xavier et al. demonstrated that exosomal chitinase 3-like-1 (CHI3L1) and fibronectin (FN) derived from anti-inflammatory macrophages induce GEM resistance via ERK/β-catenin signaling (Table 1A). Administration of pentoxifylline (PTX, a CHI3L1 inhibitor) and pirfenidone (PF, an FN inhibitor) restored PDAC sensitivity to GEM [71]. Notably, PTX and PF have been prescribed for hemorheological disorders and pulmonary fibrosis, respectively [72,73]. This highlights drug repurposing as a feasible strategy to synergistically improve GEM treatment. Binenbaum and associates showed that macrophage-derived exosome (MDE)-delivered miR-365 enhances chemoresistance through the activation of cytidine deaminase, which metabolizes GEM into its inactive form (Table 1C). MDEs play a role in GEM resistance, and Rab proteins are important in the cargo packaging, budding, docking, and fusion of sEVs in secretory and recipient cells; furthermore, a Rab27a^−/−^b^−/−^ (Rab27KO) mouse model showed a better response to GEM because of impaired exosome packaging and secretion [74]. Cancer-associated fibroblasts (CAFs) located in the stroma facilitate desmoplasia and are also involved in several cancer processes, including chemoresistance [75]. It has been reported that exposure to GEM promotes exosomal miR-146a and Snail mRNA release from CAFs, resulting in chemoresistance, which is reversed by GW4864, a blocker of exosome biogenesis and secretion [76] (Table 1C).

A previous study indicated that prolonged treatment with GEM contributes to miR-155 upregulation and exosomal miR-155 delivery to surrounding PDAC cells, which induces GEM resistance through downregulation of tumor-protein-p53-inducible nuclear protein 1 (TP53INP1), accompanied by increased antiapoptotic activity [77] (Table 1C). Recent evidence has demonstrated the ability of lncRNAs and circRNAs to sponge miRNAs, which influences the ability of miRNAs to target mRNAs and regulate protein expression [58,59,78]. In PDAC, the lncRNA SBF2-AS1 binds with miR-142-3p to induce GEM resistance through upregulation of twinfilin 1 [79]. Yin et al. noted that M2-macrophage-derived exosomal SBF2-AS1 represses miR-122-5p, which leads to reduced GEM efficacy through upregulation of X-linked inhibitor of apoptosis protein (XIAP) [80] (Table 1D). Collectively, exosomal cargos play a crucial role in PDAC resistance to GEM, greatly worsening patient prognosis. Administration of GEM combined with agents targeting exosomal contents will likely improve treatment efficacy, but further clinical exploration is needed.

### 3.2. Angiogenesis

Angiogenesis refers to vascular growth from preexisting blood vessels and is defined as a hallmark of cancer. In general, angiogenesis is involved in tumor progression and the development of metastasis [81,82]. For instance, a previous study revealed that microvessel density (MVD) positively correlates with PDAC progression [83]. Accumulating evidence suggests that PDAC angiogenesis can be facilitated through exosomal cargos within the TME. Yang et al. suggested that M2-macrophage-derived exosomal miR-155-5p and miR-221-5p promote angiogenesis via downregulation of E2F transcription factor 2 (E2F2), which impedes angiogenesis in mouse aortic endothelial cells (Table 1C). Furthermore, exosome treatment has been shown to cause an increase in MVD and tumor weight in vivo [84]. PDAC is characterized by hypoxic conditions, mainly resulting from rapid growth, dense stroma, and hypovascularity, which results in tumor progression [85]. Guo and colleagues demonstrated that uptake of hypoxic PDAC-cell-derived exosomal lncRNA UCA-1 by human microvascular vein endothelial cells induces angiogenesis. Mechanistically, UCA1 acts as a sponge for miR-96-5p, subsequently upregulating angiomotin-like protein 2 (AMOTL2), which is required for endothelial cell polarization and migration, as well as inducing ERK1/2 signaling (Table 1D). Furthermore, elevated expression of exosomal UCA-1 is correlated with factors related to poor clinical outcomes, including lymphatic invasion and advanced TNM stage [86,87]. Another study also indicated that exosomes enriched in miR-30b-5p from hypoxic PDAC cells promote tube formation via direct downregulation of gap junction protein α1 (GJA1) (Table 1C). Serum exosomal miR-30b-5p is capable of discriminating PDAC patients from HSs [88]. Of note, the role of angiogenesis in PDAC progression remains controversial. Previous studies have reported that angiogenesis plays a role in cancer progression, while the efficacy of antiangiogenic agents (e.g., anti-VEGF inhibitors such as sorafenib) in PDAC has not been as promising as that in metastatic colorectal cancer and breast cancer [89,90]. This might be explained by the strong desmoplastic reaction and hypovascularity of PDAC. ECM-deposition-mediated interstitial fluid pressure compromises vascular growth, subsequently blocking the infiltration of immune effector cells and chemotherapeutic agents [82,91]. In addition, the development of mature blood vessels indicates a better prognosis in PDAC patients due to the elevated accumulation of memory CD4^+^ T cells along with cytotoxic T cells [91]. Overall, the distinct mechanism of angiogenesis in PDAC development requires further investigation, but exosomal miRNAs and lncRNAs might serve as diagnostic tools and drug targets to systemically improve PDAC patient prognosis.

### 3.3. Invasion and Metastasis

Metastasis is a complex and multistep process that includes local invasion, intravasation, travel through circulation, extravasation, and colonization [81]; it is also the main cause of cancer-associated mortality [92]. PDAC is characterized by early progression in the form of distant metastasis [93], and the establishment of a premetastatic niche is frequently observed in early stages, even in premalignant stages [94]. Hepatic stellate cells (HSCs) are physiologically responsible for ECM homeostasis; under environmental stimuli, they transdifferentiate into myofibroblasts and become activated to promote ECM deposition, resulting in fibrosis and facilitating metastasis [95,96,97]. For instance, macrophages residing in the liver have been found to secrete granulin to transform HSCs into myofibroblasts that produce periostin (POSTN) to drive liver metastasis [98]. Accumulating studies have suggested that primary PDAC cells can modulate HSCs to facilitate liver metastasis via PDAC-cell-derived EVs. Bruno et al. reported that the PDAC-cell-derived exosome protein cargo macrophage migration inhibitory factor (MIF) can travel to the liver, where it stimulates HSCs to secrete FN (Table 1A). Elevated levels of FN recruit macrophages and neutrophils to establish the liver premetastatic niche [99]. Xie and colleagues demonstrated that exosomal CD44v6/C1QBP activated HSCs by phosphorylation of insulin-like growth factor 1, resulting in ECM remodeling to facilitate liver metastasis (Table 1A). Clinically, the expression of CD44v6/C1QBP is significantly higher in postoperative PDAC patients with liver metastasis [100]. Similarly, POSTN derived from pancreatic stellate cells (PSCs) promotes PDAC invasion through exosomal lncRNA LINC01133-induced Wnt/β-catenin signaling (Table 1D). In vivo, markedly increased tumor growth and peritoneal metastasis were noted after administration of exosomal LINC01133 [101].

M2 macrophages are widely acknowledged as playing a role in immunosuppression, and emerging studies have revealed their involvement in metastasis. It is evident that under hypoxic conditions, PDAC-cell-derived exosomal miR-301a-3p promotes M2 polarization and subsequently enhances metastatic capacity through M2 macrophage release of TGF-β, IL-10, and arginase [102] (Table 1C). Linton et al. suggested that exosomal arachidonic acid from PDAC ascites induced M2 polarization and subsequent secretion of MMP-9, promoting tumor invasion [103] (Table 1B). Another study reported that M2-macrophage-derived exosomal miR-501-3p promotes liver and lung metastasis through inhibition of TGF-beta receptor III and activation of TGF-β signaling [104] (Table 1C). As mentioned previously, lncRNAs and circRNAs function as sponges to impede several miRNA functions and induce metastasis. Li and colleagues indicated that the PDAC-cell-derived exosomal lncRNA Sox2 overlapping transcript (Sox2ot) promotes liver metastasis. Mechanistically, Sox2ot serves as a competing endogenous RNA (ceRNA) that directly binds miR-200c to induce Sox2, which causes the transformation of PDAC cells into a mesenchymal-like phenotype [105] (Table 1D). Exosomal circ-PDE8A from liver-metastatic PDAC cells functions as an miRNA sponge to sequester miR-388, which upregulates metastasis associated with colon cancer 1 (MACC1) and further activates the MET/ERK signaling pathway to enhance lymphatic, vascular, and duodenal invasion [62] (Table 1E). Another study conducted by Li suggested that PDAC-cell-derived exosomal circ-IARS acts as a sponge for miR-122 and regulates the permeability of the endothelial monolayer to enhance invasion and metastasis (Table 1E). Furthermore, elevation of exosomal circ-IARS is related to metastatic disease and shorter postoperative survival time [63].

Notably, specific exosomal contents are potential biomarkers for monitoring disease progression. Takahashi and colleagues reported that the PDAC-cell-derived exosomal lncRNA highly upregulated in liver cancer (HULC)-triggered invasion (Table 1D); additionally, the level of exosomal HULC was superior in discriminating PDAC patients from HSs compared to serum CA19-9 [106]. Another study reported that elevated levels of exosomal AFAP1-As1 correlated with local recurrence and metastasis after resection [60] (Table 1D). Li et al. revealed that highly invasive PDAC-cell-derived exosomal miR-222 induced the metastasis of low-grade malignant cells by suppressing p27 translocation into the nucleus (Table 1C). Elevated levels of plasma exosomal miR-22 are correlated with advanced TNM stages and can function as an independent prognostic indicator [107]. Apart from their utility as prognostic biomarkers, exosomes also seem to be promising exogenous miRNA delivery systems for cancer treatment. Upregulation of mothers against decapentaplegic homolog 3 (SMAD3) is reported to induce EMT in PDAC and correlates highly with LN metastasis and a shorter time to recurrence [108]. Ding and associates engineered exosomes derived from human umbilical cord mesenchymal stromal cells (hucMSCs) and incorporated exogenous miR-145-5p; these exosomes were capable of inhibiting PDAC invasion via suppression of Smad-3/TGF-β-mediated EMT. In vivo, PDAC-bearing mice displayed decreased metastasis and a lower risk of early recurrence after engineered exosome treatment [109]. However, the efficacy in clinical applications requires further investigation.

### 3.4. Immune Surveillance Escape

Due to their remarkable success in curing hematological malignancies, immune checkpoint inhibitors (ICIs) have opened a new era for cancer therapeutics [110]. Nevertheless, ICI efficacy is reportedly poor in patients with PDAC, which can be attributed to an extremely immunosuppressive TME [111,112]. Dendritic cells (DCs), important antigen-presenting cells (APCs), play a significant role in bridging innate and adaptive immunity. Type I conventional DCs (cDC1s) are professional APCs that participate in T-cell priming and tumor-associated antigen presentation. A previous study reported that cDC1 dysregulation is observed in pancreatic intraepithelial neoplasias (PanINs), with PanIN cells secreting IL-6 into the circulation, systemically inducing aberrant cDC1s [113]. Interestingly, recent evidence suggests that PDAC-derived EVs target DCs to compromise their functions and result in immune escape. According to Ding et al., elevated exosomal miR-212-3p derived from PDAC cells can be taken up by DCs, downregulating MHC II expression and further suppressing CD4^+^ T-cell activation via inhibition of regulatory factor X-associated protein (RFXAP), a transcription factor of MHC II [114] (Table 1C). Furthermore, exosomal miR-203 taken up by DCs downregulates TLR-4 accompanied by reduced expression of TNF-α and IL-12, causing dysregulation of DC-mediated immunity [115] (Table 1C). According to the study of Chen et al., DCs were found to have a lower ability to stimulate CD4^+^ and CD8^+^ T cells after administration of the PDAC-cell-derived exosomal lncRNA ENST00000560647, triggering DC-mediated immunosuppression [116] (Table 1D). The rate of response to ICIs is determined by several factors, such as tumor mutation burden, functions of immune effector cells, and proximity of cytotoxic T cells to tumors [112,117]. As mentioned previously, DCs play important roles in bridging innate and adaptive immunity, including activating cytotoxic T cells; hence, targeting exosome-mediated DC suppression might improve ICI efficacy and simultaneously enhance immunosurveillance.

**Table 1:** Involvement of exosomal cargos in PDAC progression.

Exosomal Content	Recipient Cell	Targeted Cell	Effect/Mechanism	References
**A. Protein**				
CD44v6/C1QBP complex	PDAC cell	HSC	Facilitate liver metastasis through ECM remodeling	[100]
CHI3L1 and FN	M2 macrophage	PDAC cell	Induce GEM resistance via the ERK/β-catenin signaling pathway	[71]
MIF	PDAC cell	Kupffer cell	Establish the liver premetastatic niche through FN	[99]
**B. Lipid**				
Arachidonic acid	Metastatic PDAC cell	Macrophage	Induce secretion of MMP-9 to promote tumor invasion	[103]
**C. miRNA**				
miR-146a	CAF	PDAC cell	Enhance GEM resistance	[76]
miR-155	PDAC cell	PDAC cell	Induce GEM resistance via TP53INP1 suppression	[77]
miR-155-5p and miR-221-5p	M2 macrophage	PDAC cell	Promote angiogenesis via downregulation of E2F2	[84]
miR-203	PDAC cell	DC	Induce DC dysregulation via TLR-4 downregulation	[115]
miR-212-3p	PDAC cell	DC	Induce MHC II downregulation via inhibition of RFXAP	[114]
miR-222	Highly invasive PDAC cell	PDAC cell with low malignancy	Induce metastatic capability via p27 phosphorylation	[107]
miR-301a-3p	Hypoxic PDAC cell	Macrophage	Induce M2 polarization and enhance metastatic capacity	[102]
miR-30b-5p	Hypoxic PDAC cell	EC	Promote angiogenesis via downregulation of the gap junction protein GJA1	[88]
miR-365	M2 macrophage	PDAC cell	Enhance GEM resistance through activation of cytidine deaminase	[74]
miR-501-3p	M2 macrophage	PDAC cell	Promote metastasis through activation of TGF-β signaling	[104]
**D. lncRNA**				
ENST00000560647	PDAC cell	DC	Trigger DC-mediated immunosuppression	[116]
HULC	PDAC cell	PDAC cell	Promote invasion	[106]
LINC01133	PSC	PDAC cell	Induce peritoneal metastasis via Wnt/β-catenin signaling	[101]
SBF2-AS1	M2 macrophage	PDAC cell	Induce GEM resistance via miR-122-5p repression and XIAP upregulation	[80]
Sox2ot	Highly invasive PDAC cell	PDAC cell	Promote liver metastasis via miR-200c repression and Sox2 upregulation	[105]
UCA-1	Hypoxic PDAC cell	EC	Enhance angiogenesis via the miR-96-5p/AMOTL2/ERK1/2 axis	[86,87]
**E. circRNAs**				
Circ-IARS	PDAC cell	EC	Enhance invasion and metastasis via miR-122 repression	[63]
Circ-PDE8A	Liver-metastatic PDAC cell	PDAC cell with low malignancy	Induce duodenal invasion via the miR-338/MACC1/MET axis	[62]

Abbreviations: AMOTL2: angiomotin-like protein 2; CAFs: cancer-associated fibroblasts; CHI3L1: exosomal chitinase 3-like-1; DC: dendritic cell; E2F2: E2F transcription factor 2; EC: endothelial cell; ECM: extracellular matrix; FN: fibronectin; GEM: gemcitabine; GJA1: gap junction protein α1; HSCs: hepatic stellate cells; HULC: highly upregulated in liver cancer; MACC1: metastasis associated with colon cancer 1; MIF: migration inhibitory factor; RFXAP: regulatory-factor-X-associated protein; Sox2ot: Sox2 overlapping transcript; TP53INP1: tumor protein p53 inducible nuclear protein 1; XIAP: X-linked inhibitor of apoptosis protein.

## 4. Exosomes as Biomarkers for Early Detection and Prognosis Prediction

CA19-9 has come to be recognized as a screening biomarker for PC in the clinic over the past few decades [118]. However, serum CA19-9 levels are not applicable for early detection and are unable to discriminate between PC and benign disease (e.g., chronic pancreatitis) or other malignancies, such as bile duct and gastrointestinal tract disorders [119]. According to statistics, the sensitivity and specificity of serum CA19-9 levels for the diagnosis of PC are 79–81% and 82–90%, respectively [120]. Unfortunately, CA19-9 is a modified Lewis (a) blood group antigen; therefore, individuals who are Lewis negative, accounting for 10% of the population, do not express CA19-9, leading to false-negative outcomes [119]. Hence, a more effective biomarker to improve early detection and prognosis is urgently needed. Because exosomes are secreted by parental cells, can deliver intracellular signals, and are detectable in small amounts of body fluid, they are promising biomarkers for early detection and prognostication [9,15].

Glypican-1 (GPC1), a cell surface heparan sulfate proteoglycan, is specifically detected on PDAC-cell-derived exosomes but not on nontumor-cell-derived exosomes [121]. An investigation of clinical samples by Lu et al. found that the mRNA and protein expression of GPC1 is significantly enriched in PDAC tissues compared to normal tissues. Elevated ectopic GPC1 expression corresponded to large tumor diameters and poor overall survival, indicating that it is a prospective prognostic marker for PDAC [122]. In addition, mutant *KRAS* mRNA in circulating GPC1^+^ exosomes in serum is detectable even with negative (MRI) findings and a lack of obvious pancreatic lesions, suggesting that it can detect premalignant lesions [11]. Notably, *KRAS* mutation is common in PDAC patients, with a 90% frequency, and mutated *KRAS* is reported to promote PanINs, indicating its role in the early onset of PDAC [123,124]. Conversely, a small number of studies have claimed that GPC1 may not be an ideal diagnostic marker for PDAC. For instance, a comparative analysis of exosomal GPC1 and miRNA in circulatory exosomes of healthy, chronic pancreatitis, and PDAC participants revealed that exosomal miRNA showed better differentiation of normal, CP, and PDAC patients than GPC1 [125]. Another study demonstrated that PDAC tumors with high GPC1 expression had GPC1-enriched circulating exosomes (cirExos); however, GPC1 levels in cirExos could not be used to differentiate between PDAC and benign pancreatic disease [126]. Interestingly, GPC1-enriched cirExos declined following PDAC resection, and a high cirExos GPC1 level was associated with a large tumor size, indicating that GPC1 might be associated with tumor burden and might not be a PDAC biomarker. Nevertheless, a larger number of studies advocate GPC1 as a specific surface marker on PDAC-cell-derived exosomes, suggesting that further detailed investigations of the utility of GPC1 in diagnostic or prognostic strategies are warranted.

Recently, liquid biopsies have attracted increasing attention with the advancement of precision medicine in cancer therapeutics [127]. In particular, recent investigations have focused mainly on circulating free DNA (cfDNA) and circulating tumor DNA (ctDNA), bridging liquid biopsies and personalized medicine. Previous studies have also suggested that cfDNA can serve as a marker to identify *KRAS* mutations and that ctDNA combined with serum protein biomarkers can be assessed as an early diagnostic marker in PDAC patients [128,129]. Nevertheless, there are several advantages of exosomes over cfDNA and ctDNA. (1) Exosomes are stable during storage due to their bilayer structure, and repeated freezing or thawing minimally affects their bioactivity. This prevents exosomal mutant DNA or RNA from being degraded [130]. (2) cfDNA and ctDNA are produced from dying cells (e.g., necroptotic or apoptotic cells) and are thus not accessible for real-time assessment of cellular biological conditions. Conversely, exosomes are present and can be detected during the early stage of disease onset. For instance, Allenson et al. reported that mutant *KRAS* exosomal DNA is a more specific early stage biomarker than mutant *KRAS* cfDNA. In a discovery cohort, *KRAS* mutations in exosomal DNA were detected in 7.4%, 66.7%, 80%, and 85% of healthy individuals, patients with localized disease, patients with locally advanced disease, and patients with metastasis, respectively; however, mutant *KRAS* cfDNA was detected in 14.8%, 45.5%, 30.8%, and 57.9% of these individuals, respectively [131]. This finding indicates that exosomal DNA is superior to cfDNA for distinguishing between patients with disease and healthy individuals. (3) The serum level of mutant cfDNA is low, especially in early stage malignancies; unfortunately, nonmutant cfDNA and mutant cfDNA coexist in serum, which results in a poor signal-to-noise ratio [132]. On the other hand, in Melo’s study, mutant *KRAS* transcripts were exclusively detected in GPC1^+^ exosomes, significantly improving the signal-to-noise ratio [11,15]. Nevertheless, cfDNA is superior to exosomal DNA in several ways and can serve as a powerful diagnostic tool. A standardized and automatic method for cfDNA analysis has been established; however, no such method has been established for exosomal DNA analysis [132].

Lab-on-a-chip is an approach for developing microfluidic-based microfabricated devices with exosomal physiochemical properties [133]. Indeed, with significant progress in microfluidics research, the use of chip-based assays for exosome isolation has gained popularity on the basis of its advantages over conventional exosome isolation (e.g., ultracentrifugation) because of their superiority in terms of sensitivity, low sample volume requirement, rapid processing, low reagent consumption, and cost effectiveness [134]. Lewis’s group developed an alternative current microarray chip to detect and analyze exosomal proteins via on-chip immunofluorescence, which achieved 99% sensitivity and 82% specificity in distinguishing PDAC patients from HSs. The low sample volume requirement (approximately 25 μL) and completion within 30 min are benefits that may meet clinical demands in the future [135]. Although developing a microfluidic platform for exosome detection seems promising because it would allow the integration of a multistep process on a chip, there are still several difficulties that require further investigation. Immunoaffinity is currently used for numerous chip-based approaches of exosome isolation. Wang et al. reported that the throughput of microfluidic isolation is low and that designing a multichannel format might improve efficacy. In addition, cancer is notorious for high heterogeneity; hence, the identification of specific surface markers to distinguish the cells from which exosomes originate is paramount to avoid false-negative results. Finally, standardization of exosome isolation and detection strategies is urgently needed to enable translation of such techniques from bench to bedside [134]. Chen and associates indicated three main criteria for good manufacturing practice (GMP) of exosomes: cell cultivation, exosome isolation, and validation of exosome physiochemical properties [136]. The goal of GMP is to standardize operating procedures and parameters across different platforms to optimize exosome processing, but much additional development is needed.

## 5. Discussion and Future Perspectives

As mentioned above, the dismal survival rate of PDAC is attributed to late diagnosis. Although the CA19-9 level has been employed in clinical practice for decades, it cannot be used to detect early stage or even premalignant stage PDAC or to effectively discriminate between PDAC and non-PDAC individuals [118,119]. Hence, an improved screening model is urgently needed. It is estimated that the incidence of PDAC is approximately 13.1 per 100,000 persons [137]. Thus, large-scale screening would lead to false-positive cases, and there could be unnecessary increases in patient concern and public health expenditures if these patients undergo further CT or MRI investigation [138]. In general, regular screening necessitates the selection of high-risk populations (e.g., identified with novel biomarkers). Data suggest that patients diagnosed with new-onset diabetes (NOD) and aged over 50 years have an eightfold higher risk of developing PC [139]. Hence, Sharma and colleagues established a model (the Enriching New-Onset Diabetes for Pancreatic Cancer, abbreviated as ENDPAC, model) to identify the risk of developing PC in a subset of patients with NOD on the basis of three risk factors: age, weight loss, and blood sugar elevation; in this model, classification as low (≤0), intermediate (1–2), or high (≥3) risk depends on the total score [140]. The authors claimed that patients with a score ≤0 can be managed as T2-NOD patients, with no further medical examination warranted due to the high negative predictive value; in contrast, those patients in the intermediate- or high-risk group should undergo continuous clinical work-up [140]. Circulating GPC1^+^ exosomes containing mutant *KRAS* mRNA or DNA are detectable in early stage PDAC [11]. In particular, *KRAS* mutation is involved in early pancreatic carcinogenesis at premalignant stages [141]. Consequently, intermediate- or high-risk NOD patients are candidates for exosome analysis, which will enhance early detection.

Apart from exosomes, an increasing number of extracellular vesicles and particles have been identified in recent years. One salient example is supermeres (<50 nm) isolated from the supernatant of exomeres and nonmembranous nanoparticles. Exosomes and macrophages differ in size, morphology, and cellular uptake kinetics. Nevertheless, several clinically significant protein and miRNA markers enriched in exosomes are also observed in supermeres. Moreover, the enrichment of cargo proteins is higher in supermeres than in exosomes [142,143]. This highlights that supermeres are also promising candidates for detection in liquid biopsies. However, there are several limitations that urgently need further investigation. For instance, high-density lipoprotein also transports miRNA; hence, efficient purification of supermere miRNA is a major challenge [142].

Mounting evidence suggests that the dense stroma surrounding the tumor compartment greatly contributes to drug resistance in PDAC, mediated by poor perfusion and physical barriers that compromise drug entry [91,144]. As such, exosomes are a valuable tool for PDAC treatment for two main reasons. (1) Exosomes easily overcome impenetrable barriers, such as the blood–brain barrier and dense stroma [145,146]. (2) Exosomes have low immunogenicity compared to other nanoparticle vesicles because they are natural endogenous nanovesicles and thus do not induce systemic allergic reactions [147]. Zhou and associates designed exosomes to reprogram the PDAC TME into an immunostimulatory TME in vitro and in vivo. Gal-9 is highly expressed, and its interaction with dectin-1 triggers M2 polarization [148]; hence, in this study, Gal-9 siRNA and oxaliplatin (OXA) prodrugs were encapsulated in exosomes derived from bone marrow mesenchymal stem cells. OXA facilitates immunogenic cell death to release high-mobility group box 1 (HMGB1) and ATP, promoting DC maturation and antigenic presentation. Furthermore, these designed exosomes induced CD8^+^ T-cell infiltration and M1 polarization [145]. Another study conducted by Zhou demonstrated that exosomes loaded with PTX and dFdCMP, an intermediate of GEM, had superior penetration and antitumor effects. Notably, mild systemic toxicity occurred after the administration of therapeutic exosomes in vivo [146]. Moreover, as of 2020, several pharmaceutical companies are developing therapeutic exosomes, with several deals amounting to over billions of dollars [149]. Collectively, engineering exosomes to deliver candidate drugs seems to be a promising strategy to improve antitumor drug accumulation.

**Figure 2:**
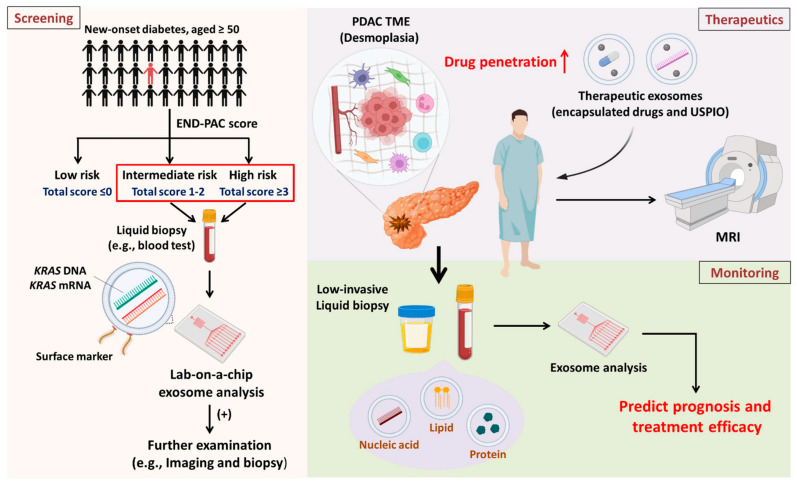
Overview and future perspectives **Screening:** Patients diagnosed with new-onset diabetes and aged ≥50 years are eight times more likely to develop PDAC. The high-risk population can be classified into three subpopulations according to the ENDPAC score. It is suggested that high-risk and intermediate-risk subpopulations receive regular blood tests for exosomal analysis to identify patients suspected of having PDAC early for further examination. **Therapeutics:** PDAC is characterized by desmoplasia, which compromises drug entry into malignant lesions. Drug candidates and USPIOs can be encapsulated in exosomes to improve drug entry and enable the detection of drug distribution via noninvasive imaging approaches. **Monitoring:** After treatment, it is important to investigate therapeutic efficacy. Analyzing exosome contents by liquid biopsies (which have low invasiveness) can provide physicians with immediate information about patient prognosis and response to treatment.

In addition to engineering therapeutic exosomes, tracking exosome distribution in vivo is important. Preliminary studies have indicated that magnetic particle imaging (MPI) or MRI combined with superparamagnetic iron oxides (SPIOs) has potential for noninvasive imaging [150]. For instance, Rivera-Rodriguez embedded adoptive T cells with ferucarbotran, a clinically approved SPIO, ex vivo and evaluated the biological distribution and persistence of injected adoptive T cells in recipients [150]. Owing to the limited size of exosomes, standard SPIOs (>50 nm) are not applicable for direct labeling and tracking of exosomes. Therefore, ultrasmall SPIOs (USPIOs, 10–50 nm) are considered an appropriate candidate for tagging therapeutic exosomes. Hu and colleagues loaded melanoma-derived exosomes with SPION5 (4.5 nm) via electroporation, which facilitated the detection of exosome trafficking in vivo under MRI [151] and demonstrated that USPIOs taken up by bone marrow mesenchymal stromal cells were transported to and colocalized with intracellular vesicles expressing CD9, CD63, and CD81, indicating that exosomes secreted from parental cells contain deposited USPIOs [152]. Additionally, ferumoxytol is gaining popularity over ferucarbotran in clinical studies. Ferumoxytol has received FDA approval and is a safe magnetic nanoparticle (NP) that can be used to improve the detection of LNs for clinical cancer staging in patients with primary prostate or breast cancer (NCT00087347). Makela et al. reported that ferumoxytol NPs are superior to ferucarbotran in the detection of iron-labeled TAMs [153]. Importantly, ferumoxytol exerts low toxicity toward recipient cells and is a USPIO [154]. In summary, loading therapeutic exosomes with ferumoxytol appears to be a promising strategy to evaluate whether injected exosomes penetrate and accumulate within malignant lesions. Such tools can provide physicians with real-time information about the in vivo distribution of therapeutic exosomes (Figure 2).

Although exosomes have the potential to serve as biomarkers for early detection and drug carriers for PDAC therapeutics, there are several limitations regarding clinical implications that warrant further investigation. First, standardization of exosome isolation and detection is urgently needed because the procedure varies among different laboratories and experimental designs. Second, ultracentrifugation remains the gold standard for exosome isolation, but its efficiency is poor, making it difficult to meet clinical demands [155]. Third, storage is a major concern. Gelibter et al. evaluated several factors (e.g., cargo concentration, purity, and size) of exosomes after −80 °C storage and freeze-thaw cycles. It was observed that size increased with variability, purity decreased, and cargo loads decreased in a time-dependent manner. Furthermore, the fusion phenomenon occurred after freeze-thaw cycles [156]. Notably, the protein concentration in the supernatant increased, possibly due to particle lysis as well as protein leakage from exosomes [157]. Finally, safety concerns must be considered, even if repeated injection of mesenchymal stem cell (MSC)-derived exosomes (MDEs) into immunocompetent mice led to minimal inflammation and MDEs were well tolerated in patients with refractory graft-versus host disease [130,158]. Previous findings have indicated that MDEs as therapeutics promoted cancer multidrug resistance instead of suppressing tumor progression [159]. Additionally, which source of exosomes is the most suitable to serve as a drug carrier and whether preconditioning exosome-secreting cells has severe impacts on the function and efficacy of therapeutic exosomes require further investigation [160].

## 6. Conclusions

PDAC is a lethal and aggressive malignancy with a dismal five-year survival rate of 11%, mainly attributed to the lack of effective diagnostic tools for early detection. Although serum CA19-9 has been clinically applied as a tumor biomarker in PDAC screening for decades, several drawbacks, such as inferior sensitivity as well as specificity and false-negative detection in the Lewis-antigen-negative population, limit the diagnostic value of serum CA19-9. Research into sEVs has become a focus in recent years. In particular, exosomes and their cargos are involved in PDAC tumorigenesis (e.g., chemoresistance, metastasis, and immunosuppression) and are detectable in body fluids. Thus, they serve as potential low-invasive biomarkers for PDAC diagnosis and disease monitoring. Furthermore, specific surface markers have been reported to efficiently differentiate PDAC patients from HSs. Although exosomes have promising clinical implications, there are several limitations and urgent problems that require in-depth investigations in the clinical setting in the future, including (1) optimizing the standardization of exosome isolation, detection, and quantification; (2) exploring more effective storage approaches, such as −80°C storage and freeze-thaw cycles, which considerably influence the quality of exosomes; and (3) evaluating the safety of exosomes if applied as drug carriers.

## Abbreviations

APCs: antigen-presenting cells; CAFs: cancer-associated fibroblasts; cDC1s: type I conventional dendritic cells; ceRNA: competing endogenous RNA; cfDNA: circulating free DNA; CHI3L1: chitinase 3-like-1; circRNA: circular RNA; CP: chronic pancreatitis; ctDNA: circulating tumor DNA; DCs: dendritic cells; ECM: extracellular matrix; ESCRT: endosomal sorting complex required for transport; FN: fibronectin; GEM: gemcitabine; GMP: good manufacturing practice; GPC1: glypican-1; HSs: healthy subjects; HSCs: hepatic stellate cells; hucMSCs: human umbilical cord mesenchymal stromal cells; ICIs: immune checkpoint inhibitors; ILVs: intraluminal vesicles; LN: lymph node; lncRNAs: long noncoding RNAs; miRNA: microRNA; MRI: magnetic resonance imaging; MVBs: multivesicular bodies; MVD: microvessel density; ncRNAs: noncoding RNAs; NOD: new-onset diabetes; NP: nanoparticle; PanIN: pancreatic intraepithelial neoplasia; PC: pancreatic cancer; PDAC: pancreatic ductal adenocarcinoma; PF: pirfenidone; PNI: perineural invasion; POSTN: periostin; PSCs: pancreatic stellate cells; PTX: pentoxifylline; RFXAP: regulatory-factor-X-associated protein; rRNA: ribosomal RNA; sEVs: small extracellular vesicles; snRNA: small nuclear RNA; SPIO: superparamagnetic iron oxide; TME: tumor microenvironment; TP53INP1: tumor protein p53 inducible nuclear protein 1; USPIO: ultrasmall superparamagnetic iron oxide; XIAP: X-linked inhibitor of apoptosis protein.
